# Ferromagnetic Resonance and Antiresonance in Composite Medium with Flakes of Finemet-Like Alloy

**DOI:** 10.3390/nano11071748

**Published:** 2021-07-02

**Authors:** Dmitry V. Perov, Anatoly B. Rinkevich

**Affiliations:** M.N. Miheev Institute of Metal Physics UB RAS, Sofia Kovalevskaya St., 18, 620108 Ekaterinburg, Russia; peroff@imp.uran.ru

**Keywords:** magnetic composites, microwaves, absorption, transmission and reflection coefficients

## Abstract

Propagation of microwaves is studied in a composite material containing flakes of Fe-Si-Nb-Cu-B alloy placed into an epoxyamine matrix. The theory is worked out, which permits to calculate the coefficients of the dynamic magnetic permeability tensor and the effective magnetic permeability of the transversely magnetized composite. The measurements of magnetic field dependences of the transmission and reflection coefficients were carried out at frequencies from 12 to 38 GHz. Comparison between calculated and measured coefficients were conducted, which show that the calculation reproduces all main features of the resonance variations caused by ferromagnetic resonance and antiresonance. The dissipation of microwave power was calculated and measured. It is shown that the penetration depth of the electromagnetic field increases under antiresonance condition and decreases under resonance.

## 1. Introduction

Composite media can have properties which are absent for their composing parts. Composite materials containing metallic particles in the dielectric matrix present important areas of application [1]. The microwave magnetic properties of composites look promising, but, so far, they have not been adequately explored. The laws governing the frequency dispersion in the magnetic composites are discussed in detail [2]. The mixing rules for the constitutive parameters are presented in the monograph [3]. The complex magnetic permeability is measured in the frequency range of 0.01–10 GHz for magnetically structured granular systems using the transmission/reflection waveguide method [4]. In order to secure a high level of electromagnetic screening, the particles in the form of flakes placed in a polymer matrix are used [5]. The composite with Fe_16_Ni_82_Mo_2_ alloy particles shows fairly good shielding results in the frequency range from 2 to 18 GHz [6]. Microwave magnetic permeability for the composites with carbonaceous particles is studied [7,8].

Resonance-type phenomena, such as ferromagnetic resonance and antiresonance, can be observed on microwaves for a ferromagnetic medium placed in a DC magnetic field. Ferromagnetic resonance (FMR), as known, leads to absorption of electromagnetic waves [9]. Phenomenon of ferromagnetic antiresonance (FMAR) has been observed earlier if electromagnetic wave transmits through a thin ferromagnetic metallic film. In this case, the FMAR condition leads to a sharp increase in the transmission of microwaves through a film [10]. This phenomenon occurs providing that the real part of the dynamic permeability equals to zero in a magnetic field lower than the FMR field [9,11]. In paper [12], the FMAR phenomenon is observed experimentally in a nanocomposite medium. The FMAR phenomenon in a composite medium demonstrates a number of specific features. For nanocomposites not having the DC conductivity, the skin effect is absent. Instead of an increase in microwave transparency of the sample, in non-conductive nanocomposites, the FMAR usually causes an increase in the reflection coefficient. Nonetheless, some features of antiresonance preserve in non-conductive nanocomposites. In particular, the FMAR realizes only at frequencies above a certain value, which depends on the saturation magnetization of a material.

Due to high magnetic permeability, the finemet-type alloys are regarded as a suitable material for radio- and microwave devices. Improved electromagnetic shielding and absorbing properties are obtained for the flake-shaped and spherical-like form of Fe_16_Ni_82_Mo_2_ alloy particles [6]. The microwave complex refraction coefficient of a composite consisting of flakes of Fe-Si-Nb-Cu-B alloy placed into epoxy resin matrix has been studied [13]. The material under consideration is a dielectric at DC and a lossy dielectric at microwave frequencies in zero magnetic fields. Near the field of ferromagnetic resonance, however, the real and imaginary parts of the complex refraction coefficient are of the same order of magnitude, such as in a conductive medium. The magnetic properties of powdered nanostructured alloy Fe_73.5_Cu_1_Nb_3_Si_13.5_B_9_ have been studied in the frequency range 0.2–10.2 GHz [14].

The investigation of magnetic resonances is carried out in this paper for the composites containing flakes of finemet-type alloy at frequencies of the centimeter and millimeter wavebands. These frequency ranges were chosen in order to observe the FMAR phenomenon. The calculation was carried out for magnetic field dependences of the microwave transmission and reflection coefficients. These coefficients were also measured in the frequency range from 12 to 38 GHz. The theory of dynamic magnetic permeability of the composite media was worked out. The complexity of this problem consists, in particular, in the necessity to take into consideration the shape, dimensions and space orientation of ferromagnetic particles. The calculation of the dynamic magnetic permeability is discussed in papers [15,16]. The dependence of the effective permeability on the fraction of magnetic component was calculated. For the periodical magnetic inclusions, averaging was realized for both magnetic field and magnetic induction within the assigned cells [17,18,19]. A proposed cluster model was used in order to determine analytically the effective magnetic permeability of a composite system when this permeability is due to generation of eddy currents in an alternating magnetic field near the percolation threshold [20]. Magnetic field dependence of the relative magnetic permeability was calculated using a nonlinear homogenization approach [21]. A model based on the Landau–Lifshitz–Gilbert equation was developed to calculate the complex permeability of magnetic particles/insulator matrix composite [22].

In this paper, a new method for the calculation of the effective magnetic permeability is offered for composite magnetic materials which contain ferromagnetic particles in the form of ellipsoid with arbitrarily directed axes. Comparison between the results of experiments and theoretical calculations is outlined for transmission and reflection of microwaves. The dissipation of microwave power was calculated and the penetration depth of the wave into the sample was also estimated.

## 2. Materials and Methods

Samples of the composites were prepared of Fe-Si-Nb-Cu-B alloy particles and the epoxyamine matrix. This matrix was chosen due to its moderate dielectric permittivity and ease of preparation of the composite. Metallic particles are in the form of flakes of the finemet-type alloy with the following composition: Fe—80.1%, Si—8.5%, Nb—8.4%, Cu—1.1%, B—1.2%, Cr—0.2%, Mn—0.1%, Ni—0.1% and Co—0.3%. As known, the nanocrystalline finemet-type alloy has high magnetic permeability [14].

**Figure 1:**
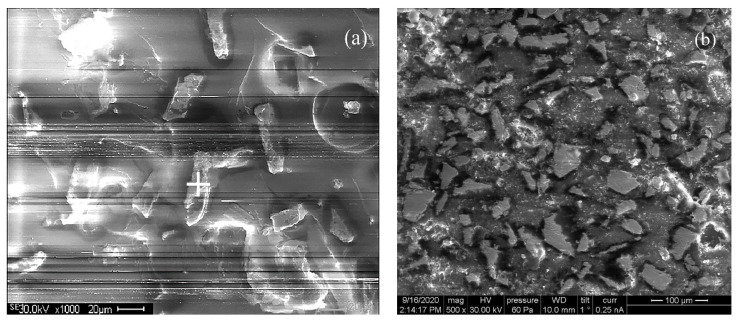
Electron microscopic images of structure of the composite with 15% flakes: inside the sample (**a**); near the top surface (**b**). Structure of the composite was studied with a Vega3 Tescan microscope at an accelerating voltage of 30 kV.

The composite material was prepared by mechanical mixing of particles in the epoxyamine matrix. After mechanical mixing, the additional mixing in the ultrasonic bath was undertaken and then hardening of the mixture occurred during several hours. Two series of composite samples were prepared with 15 wt.% and 30 wt.% particles. The X-ray phase analysis carried out with a “Pananalytical” spectrometer shows that the main phases are two BCC-type ones, with the slightly different lattice parameters of 2.871 Å and 2.841 Å. In Figure 1, structure of the composite with 15% particles is shown. The samples for microscopy were prepared both from the inner part of the sample (Figure 1a) and near the top surface (Figure 1b). It should be noted that particles were arbitrarily oriented in the inner part of sample, but some preferable orientation in parallel to the sample surface presented near the top surface. The preferable orientation of particles in the upper part of the sample appeared during the hardening process and approximately 20% of the particles had the preferable orientation in parallel to the surface, while 80% of the particles were oriented arbitrarily. The DC conductivity of the samples was very low.

**Figure 2:**
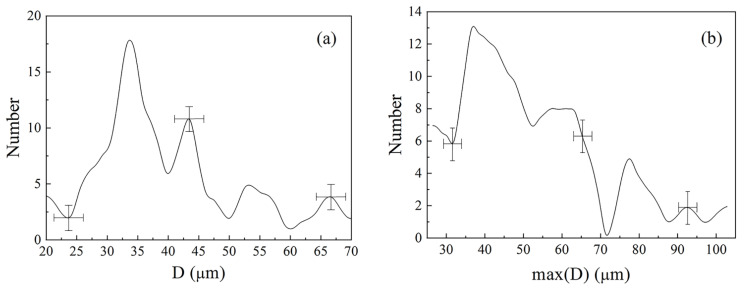
Distribution of the mean diameters of particles (**a**) and distribution of the maximal Martin diameters (**b**).

The distribution of the diameters of particles is shown in Figure 2a. The maximal Martin diameter, that is, the maximal length of a midline progressing through the particle, was 51 µm in average. The distribution of the maximal Martin diameters is shown in Figure 2b. The minimal Martin diameter was 27.4 µm in average.

**Figure 3:**
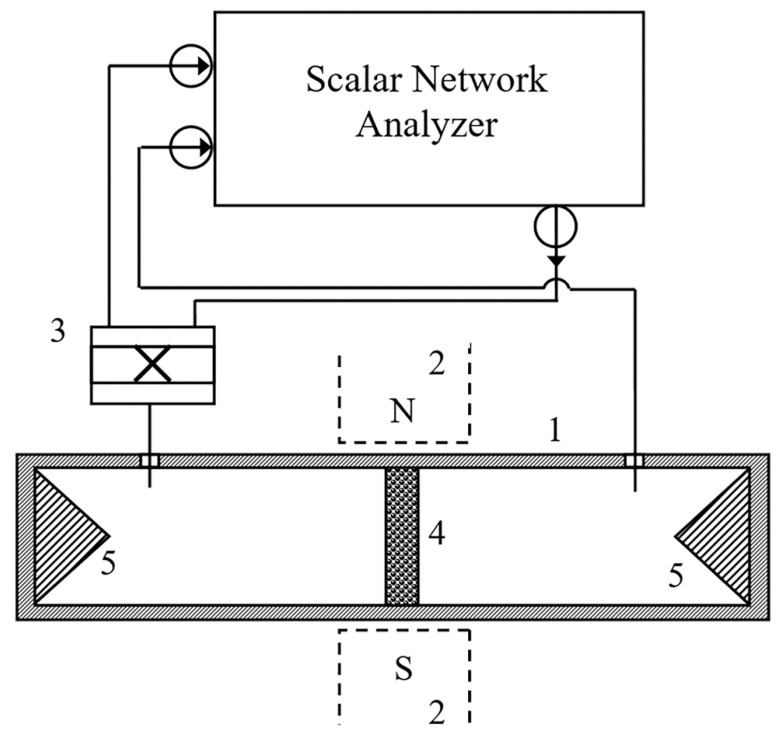
Scheme of microwave measurements: 1—waveguide, 2—electromagnet, 3—directional coupler, 4—sample and 5—absorber.

The microwave measurements were carried out at frequencies from 12 to 38 GHz, using the methods described in [12]. All microwave measurements were performed at room temperature. The scheme of the experiment is presented in Figure 3. The sample was placed in the cross-section of a rectangular waveguide 1, in order to completely overlap the cross-section. The thickness of the sample was from 1.5 to 2 mm. The waveguide operates with a TE_10_ mode and its dimensions were defined by the frequency ranges: 16 mm × 8 mm, for 12–17 GHz (WR-62); 11.5 mm × 5 mm, for 17–26 GHz (WR-42); 7.2 mm × 3.4 mm, for 27–38 GHz (WR-28). The wave fell normally to the surface of sample. The external DC magnetic field vector H lay in the plane of the plate and was directed perpendicularly to the microwave magnetic field $H_{\sim }$, $H⊥H_{\sim }$. This configuration of vectors is called transversely magnetized media.

The measurements were performed with the scalar network analyzer. The amplitudes of transmitted and reflected waves were determined with the directional couplers 2. The modules of the transmission *T* and reflection *R* coefficients and their frequency and magnetic field dependences were measured. The measurements of the coefficients in zero magnetic field were used to determine the complex dielectric permittivity $\dot{\varepsilon }=\varepsilon^{\prime }-i\varepsilon^{\prime \prime }$. The relative variations of the transmission coefficient are defined as $d_{m}=\frac{\left|T\left(H\right)\right|-\left|T\left(0\right)\right|}{\left|T\left(0\right)\right|}$, where |T(H)| is the module of transmission coefficient in the magnetic field *H*. Analogously, the relative variations of the reflection coefficient are defined as $r_{m}=\frac{\left|R\left(H\right)\right|-\left|R\left(0\right)\right|}{\left|R\left(0\right)\right|}$, where |R(H)| is the module of the reflection coefficient in the magnetic field *H*.

## 3. Results

### 3.1. Resonance Phenomena in Magnetized Composite Media

The theory of microwave propagation is here described in a transversely magnetized composite medium. On the assumption of symmetry, the tensor components of the magnetic susceptibility of a composite medium are defined. The effective magnetic parameters of the composite medium are further introduced, in which magnetic particles with form of ellipsoid are placed into a nonmagnetic dielectric material. The resonance phenomena are studied in the case of the electromagnetic wave propagation in a magnetized composite medium. The numerical calculations of the transmission and reflection coefficients, as well as their magnetic field dependences, are carried out. The magnetic field dependences of the wavenumber are drawn as well.

#### 3.1.1. Propagation of Electromagnetic Waves in Transversely Magnetized Medium

Let the electromagnetic wave propagate in an infinite macroscopically uniform medium along the 0y axis. The magnetic field **H** is directed along the 0z axis. Following to [9], let us consider the solution of Maxwell’s equations:

(1) $$\begin{array}{c} \mathrm{rot}\;e=-\frac{\partial \;b}{\partial \;t}\\ \mathrm{rot}\;h=\frac{\partial \;d}{\partial \;t} \end{array}$$

jointly with the constitutive relations:

(2) $$\begin{array}{c} d=\varepsilon_{0}\varepsilon \cdot e\\ b=\mu_{0}\overset{↔}{\mu }\cdot h \end{array}$$

where $\varepsilon_{0}$ and $\mu_{0}$ are the electric and magnetic constants, respectively. Note that the second of Equation (1) takes into account both the conduction and displacement currents when we are using the complex dielectric permittivity ε.

In Equations (1) and (2), **b** and **d** are the magnetic and electric induction and **e** and **h** are the electric and magnetic field, respectively. It is assumed that the dielectric permittivity of the medium *ε* is a scalar value and it does not depend on magnetic field. The magnetic permeability is defined by the tensor $\overset{↔}{\mu }$. At chosen directions of the fields, this tensor has a view [9]:

(3) $$\overset{↔}{\mu }=\left(\begin{array}{ccc} \mu_{xx} & \mu_{xy} & 0\\ \mu_{yx} & \mu_{yy} & 0\\ 0 & 0 & 1 \end{array}\right)$$

We will assume here that all the fields do not change in the direction of the *x* axis and the electromagnetic wave propagates along the *y* axis. Thus, the vectors **h, b, e** and **d** can be expressed in the form $h=\hat{h}\left(z\right)\exp\left[i\left(\omega t-ky\right)\right]$, $b=\hat{b}\left(z\right)\exp\left[i\left(\omega t-ky\right)\right]$, $e=\hat{e}\left(z\right)\exp{\left[i\left(\omega t-ky\right)\right]}^{}$ and $d=\hat{d}\left(z\right)\exp\left[i\left(\omega t-ky\right)\right]$, where $\hat{h}\left(z\right)$, $\hat{b}\left(z\right)$, $\hat{e}\left(z\right)$ and $\hat{d}\left(z\right)$ are the vector complex amplitude factors for the corresponding alternating fields, ω=2π f is the cyclic frequency and k is the propagation constant. The solution of the system of Equations (1) and (2) in a coordinate view is reduced to the following equations:

(4) $$\frac{\partial^{2}{\hat{h}}_{x}}{\partial \;z^{2}}-\left[\;\left(k^{2}-\varepsilon \mu_{xx}k_{0}^{2}\right)\;{\hat{h}}_{x}-\varepsilon \mu_{xy}k_{0}^{2}{\hat{h}}_{y}\right]=0$$

(5) $$\frac{\partial^{2}{\hat{h}}_{y}}{\partial \;z^{2}}-\left[\;\mu_{yx}\left(k^{2}-\varepsilon \;k_{0}^{2}\right)\;{\hat{h}}_{x}+\mu_{yy}\left(k^{2}-\varepsilon \;k_{0}^{2}\right)\;{\hat{h}}_{y}\right]=0$$

where *k* is the wavenumber in the magnetized medium and $k_{0}=\frac{\omega }{c}$, $c=\frac{1}{\sqrt{\varepsilon_{0}\mu_{0}}}$ is the speed of electromagnetic wave in vacuum. The condition of consistency of the algebraic equations systems (4) and (5) relatively to ${\hat{h}}_{x}$ and ${\hat{h}}_{y}$ is the equality to zero of the determinant of the following form:

(6) $$\det\left(\begin{array}{cc} k^{2}-\varepsilon \mu_{xx}k_{0}^{2} & -\varepsilon \mu_{xy}k_{0}^{2}\\ \mu_{yx}\left(k^{2}-\varepsilon \;k_{0}^{2}\right) & \mu_{yy}\left(k^{2}-\varepsilon \;k_{0}^{2}\right) \end{array}\right)=0$$

From here, two solutions for the wavenumbers are the following: The relation

$$k=k_{0}\sqrt{\varepsilon }$$

corresponds to the first solution. This solution is related to the wave in which the polarization of the alternating magnetic field **h** coincides with the direction of the DC magnetic field **H**. The wavenumber of this wave does not depend on the DC magnetic field. The second solution corresponds to the wave in which the alternating magnetic field is perpendicular to the magnetizing field. From (6), it follows that

(7) $$k=k_{0}\sqrt{\varepsilon \;\left(\mu_{xx}-\frac{\mu_{xy}\mu_{yx}}{\mu_{yy}}\right)}=k_{0}\sqrt{\varepsilon \;\mu_{eff}}$$

where the effective magnetic permeability is introduced as:

(8) $$\mu_{eff}=\mu_{xx}-\frac{\mu_{xy}\mu_{yx}}{\mu_{yy}}$$

Equation (8) can be used for both the uniform magnetic medium and the composite medium. The following relations are valid for the classical Polder tensor: $\mu_{xx}=\mu_{yy}=\mu$, $\mu_{xy}=i\mu_{a}$ and $\mu_{yx}=-i\mu_{a}$. Equation (8) takes the known view in this case [9]:

(9) $$\mu_{eff}=\mu -\frac{\mu_{a}^{2}}{\mu }$$

#### 3.1.2. Propagation Tensor of Magnetic Permeability of the Media with a Single Particle

Before turning to the composite medium, let us consider an alone magnetic particle in the non-magnetic medium. Assume that the particle has the form of an ellipsoid with arbitrarily directed axes in relation to the DC and microwave fields. Denote $H_{i}$ the DC magnetic field inside the ellipsoid and $H_{i}$ differs from the external magnetic field **H**. The connection between these fields is given by:

(10) $$H_{i}=H-\overset{↔}{N}M$$

where **M** is the magnetization and $\overset{↔}{N}$ is the tensor of the demagnetizing factors. This tensor, written for any particle, defines the difference of the magnetic field $H_{i}$ inside it from the outer magnetic field **H** in the surrounding non-magnetic matrix. Its off-diagonal elements obey to the following conditions: $N_{ij}=N_{ji}$ at i≠j, i, j↔x, y, z; the diagonal ones to the other condition: $N_{xx}+N_{yy}+N_{zz}=4\pi$, i.e., $\mathrm{tr}\overset{↔}{N}=4\pi$.

Let us write the Landau–Lifshitz equation for the lossy magnetic medium [9]:

(11) $$i\omega \;m+\gamma \mu_{0}\;m\times \left(H-\overset{↔}{N}M\right)+\gamma \mu_{0}\left(\overset{↔}{N}m\right)\times M+\frac{i\alpha \omega }{M}m\times M=-\gamma \mu_{0}\;M\times h$$

where M is the length of the DC magnetization vector M, h and **m** are the alternating magnetic field and magnetization, α is the dissipation parameter and γ is the gyromagnetic ratio. Equation (11) can be rewritten as:

(12) $$\left\{ \begin{array}{l} a_{11}m_{x}+a_{12}m_{y}+a_{13}m_{z}=-\gamma \mu_{0}M_{y}h_{z}+\gamma \mu_{0}M_{z}h_{y},\\ a_{21}m_{x}+a_{22}m_{y}+a_{23}m_{z}=-\gamma \mu_{0}M_{z}h_{x}+\gamma \mu_{0}M_{x}h_{z},\\ a_{31}m_{x}+a_{32}m_{y}+a_{33}m_{z}=-\gamma \mu_{0}M_{x}h_{y}+\gamma \mu_{0}M_{y}h_{x}, \end{array}\right.$$

with the following elements of the matrix $a_{ij}$:

$$a_{11}=i\frac{\omega }{\gamma \mu_{0}}+N_{xy}M_{z}-N_{xz}M_{y}$$

$$a_{12}=H_{z}+\frac{i\alpha \omega }{\gamma \mu_{0}M}M_{z}-N_{xz}M_{x}-2N_{yz}M_{y}-\left(N_{zz}-N_{yy}\right)M_{z}$$

$$a_{13}=-{\left[H_{y}+\frac{i\alpha \omega }{\gamma \mu_{0}M}M_{y}-N_{xy}M_{x}-\left(N_{yy}-N_{zz}\right)M_{y}-2N_{yz}M_{z}\right]}^{}$$

$$a_{21}=-{\left[H_{z}+\frac{i\alpha \omega }{\gamma \mu_{0}M}M_{z}-2N_{xz}M_{x}-N_{yz}M_{y}-\left(N_{zz}-N_{xx}\right)M_{z}\right]}^{}$$

$$a_{22}=i\frac{\omega }{\gamma \mu_{0}}+N_{yz}M_{x}-N_{xy}M_{z}$$

$$a_{23}=H_{x}+\frac{i\alpha \omega }{\gamma \mu_{0}M}M_{x}-\left(N_{xx}-N_{zz}\right)M_{x}-N_{xy}M_{y}-2N_{xz}M_{z}$$

$$a_{31}=H_{y}+\frac{i\alpha \omega }{\gamma \mu_{0}M}M_{y}-2N_{xy}M_{x}-\left(N_{yy}-N_{xx}\right)M_{y}-N_{yz}M_{z}$$

$$a_{32}=-\left[H_{x}+\frac{i\alpha \omega }{\gamma \mu_{0}M}M_{x}-\left(N_{xx}-N_{yy}\right)M_{x}-2N_{xy}M_{y}-N_{xz}M_{z}\right]$$

$$a_{33}=i\frac{\omega }{\gamma \mu_{0}}+N_{xz}M_{y}-N_{yz}M_{x}$$

The solution of Equation (12) lets one disclose the connection between the elements of the vectors $m={\left(\begin{array}{ccc} m_{x} & m_{y} & m_{z} \end{array}\right)}^{}$ and $h={\left(\begin{array}{ccc} h_{x} & h_{y} & h_{z} \end{array}\right)}^{}$ as $m=\overset{↔}{\chi }\cdot h$, therefore, define the elements of the magnetic susceptibility tensor:

(13) $$\overset{↔}{\chi }=\left(\begin{array}{ccc} \chi_{xx} & \chi_{xy} & \chi_{xz}\\ \chi_{yx} & \chi_{yy} & \chi_{yz}\\ \chi_{zx} & \chi_{zy} & \chi_{zz} \end{array}\right)$$

its elements have the view:

$$\chi_{xx}=\frac{M_{z}A_{21}+M_{y}A_{31}}{\det a}$$

$$\chi_{xy}=\frac{M_{z}A_{11}-M_{x}A_{31}}{\det a}$$

$$\chi_{xz}=-\frac{M_{y}A_{11}+M_{x}A_{21}}{\det a}$$

$$\chi_{yx}=-\frac{M_{z}A_{22}+M_{y}A_{32}}{\det a}$$

$$\chi_{yy}=-\frac{M_{z}A_{12}-M_{x}A_{32}}{\det a}$$

$$\chi_{yz}=\frac{M_{y}A_{12}+M_{x}A_{22}}{\det a}$$

$$\chi_{zx}=\frac{M_{z}A_{23}+M_{y}A_{33}}{\det a}$$

$$\chi_{zy}=\frac{M_{z}A_{13}-M_{x}A_{33}}{\det a}$$

$$\chi_{zz}=-\frac{M_{y}A_{13}+M_{x}A_{23}}{\det a}$$

Here, the definitions $A_{ij}$ are used. They are the minors of the matrix a, which are determined by the relation $A_{ij}=\det\left[{\left(a_{pq}\right)}_{p\neq i,q\neq j}\right]$. It is assumed, here, that $m={\left(\begin{array}{ccc} m_{x} & m_{y} & 0 \end{array}\right)}^{}$ and $h={\left(\begin{array}{ccc} h_{x} & h_{y} & 0 \end{array}\right)}^{}$.

If $H={\left(\begin{array}{ccc} 0 & 0 & H_{z} \end{array}\right)}^{}$ and $H>H_{s}$, where $H_{s}$ is the field of magnetic saturation, the inequalities $M_{x}<<M_{z}$ and $M_{y}<<M_{z}$ are valid for the projections of the vector M. Therefore, in this case, one can suppose that M||H, that is, $M={\left(\begin{array}{ccc} 0 & 0 & M_{z} \end{array}\right)}^{}$, at least at first approximation. Then the tensor (13) becomes:

(14) $$\overset{↔}{\chi }=\left(\begin{array}{ccc} \chi_{xx} & \chi_{xy} & 0\\ \chi_{yx} & \chi_{yy} & 0\\ 0 & 0 & 0 \end{array}\right)$$

$$\chi_{xx}=\frac{\omega_{M}{\left[\omega_{H}+i\omega \alpha -\left(N_{zz}-N_{yy}\right)\;\omega_{M}\right]}^{}}{D}$$

$$\chi_{xy}=\frac{\omega_{M}{\left[i\omega -N_{xy}\omega_{M}\right]}^{}}{D}$$

$$\chi_{yx}=-\frac{\omega_{M}{\left[i\omega +N_{xy}\omega_{M}\right]}^{}}{D}$$

$$\chi_{yy}=\frac{\omega_{M}{\left[\omega_{H}+i\omega \alpha -\left(N_{zz}-N_{xx}\right)\;\omega_{M}\right]}^{}}{D}$$

$$D=\left[\omega_{H}+i\omega \alpha -\left(N_{zz}-N_{xx}\right)\;\omega_{M}\right]\;\left[\omega_{H}+i\omega \alpha -\left(N_{zz}-N_{yy}\right)\;\omega_{M}\right]-{\left(N_{xy}\omega_{M}\right)}^{2}-\omega^{2}$$

where $\omega_{H}=\gamma \mu_{0}H_{z}$, $\omega_{M}=\gamma \mu_{0}M_{z}$.

Using the magnetic susceptibility tensor $\overset{↔}{\chi }$, it is possible to find the magnetic permeability tensor:

(15) $$\overset{↔}{\mu }=\overset{↔}{I}+\overset{↔}{\chi }$$

Inserting (14) into (15), one gets, according to (3):

(16) $$\overset{↔}{\mu }=\left(\begin{array}{ccc} \mu_{xx} & \mu_{xy} & 0\\ \mu_{yx} & \mu_{yy} & 0\\ 0 & 0 & 1 \end{array}\right)$$

$$\mu_{xx}=1+\frac{\omega_{M}{\left[\omega_{H}+i\omega \alpha -\left({\tilde{N}}_{zz}-{\tilde{N}}_{yy}\right)\;\omega_{M}\right]}^{}}{\tilde{D}}$$

$$\mu_{xy}=\frac{\omega_{M}\left[i\omega -{\tilde{N}}_{xy}\omega_{M}\right]}{\tilde{D}}$$

$$\mu_{yx}=-\frac{\omega_{M}\left[i\omega +{\tilde{N}}_{xy}\omega_{M}\right]}{\tilde{D}}$$

$$\mu_{yy}=1+\frac{\omega_{M}{\left[\omega_{H}+i\omega \alpha -\left({\tilde{N}}_{zz}-{\tilde{N}}_{xx}\right)\;\omega_{M}\right]}^{}}{\tilde{D}}$$

$$\tilde{D}=\left[\omega_{H}+i\omega \alpha -\left({\tilde{N}}_{zz}-{\tilde{N}}_{xx}\right)\;\omega_{M}\right]\;\left[\omega_{H}+i\omega \alpha -\left({\tilde{N}}_{zz}-{\tilde{N}}_{yy}\right)\;\omega_{M}\right]-{\left({\tilde{N}}_{xy}\;\omega_{M}\right)}^{2}-\omega^{2}$$

The designation $\overset{↔}{\tilde{N}}=\frac{1}{4\pi }\cdot \overset{↔}{N}$ is used here, at the definition $\mathrm{tr}\overset{↔}{\tilde{N}}=1$.

#### 3.1.3. Tensor of Magnetic Permeability of Composite Medium

Let us now discuss the problem of the effective magnetic parameters of the composite medium, which consists of identical magnetic particles in the form of ellipsoid with the same spatial orientation placed into the non-magnetic matrix. This composite medium is regarded as a macroscopically uniform medium, since the sizes of any elementary volume are significantly less than the characteristic scales, such as the wavelength of the propagating wave and the dimensions of the sample, but larger than the sizes of any magnetic particle. Magnetization of the composite medium is characterized by a volume fraction $\theta_{v}$ of the ferromagnetic phase, which is assumed constant for any elementary volume of the medium.

The tensor $\overset{↔}{\mu }$, in accordance with (16), takes into account the demagnetizing fields both for the DC and alternating fields. Following to [3], the expression for permeability tensor of composite ${\overset{↔}{\mu }}^{m}$ can be represented as the Silberstein formula:

(17) $${\overset{↔}{\mu }}^{m}=\left(1-\theta_{v}\right)\cdot \overset{↔}{I}+\theta_{v}\cdot \overset{↔}{\mu }$$

As is shown in [23,24], variations of the field in the composite medium at $\theta_{v}<1$ can be taken into account by introducing the effective demagnetizing tensor $\overset{↔}{\tilde{L}}$, which has the following limiting cases: at $\theta_{v}\to 0$, the approximate equality $\overset{↔}{\tilde{L}}\approx \overset{↔}{\tilde{N}}$ is valid, where the tensor $\overset{↔}{\tilde{N}}$ is defined for the single particle of a given form. At $\theta_{v}\to 1$, the effective demagnetizing tensor is close to zero, $\overset{↔}{\tilde{L}}\approx \overset{↔}{0}$, that corresponds to the unbounded magnetic medium. The paper [25] describes a mathematical model enabling the calculation of the effective permeability tensor of heterogeneous magnetic materials. The model uses a definition similar to Equation (17) and gives all the complex components of the permeability tensor. According to [23,24], the effective demagnetizing tensor can be determined as:

(18) $$\overset{↔}{\tilde{L}}=\left(\overset{↔}{\mu }-{\overset{↔}{\mu }}^{m}\right)\cdot {\left({\overset{↔}{\mu }}^{m}\cdot \left(\overset{↔}{\mu }-\overset{↔}{I}\right)\right)}^{-1}\cdot \overset{↔}{\tilde{N}}$$

Taking (17) into account, one gets the equation for $\overset{↔}{\tilde{L}}$:

(19) $$\overset{↔}{\tilde{L}}=\left(1-\theta_{v}\right)\cdot {\left(\overset{↔}{I}+\theta_{v}\cdot \left(\overset{↔}{\mu }-\overset{↔}{I}\right)\right)}^{-1}\cdot \overset{↔}{\tilde{N}}$$

Equation (19) shows that $\overset{↔}{\tilde{L}}$ is, generally speaking, dependent on $\overset{↔}{\mu }$. In the first approximation for $\theta_{v}<<1$ and $H>H_{s}$, it is reasonable to consider this dependence as negligible and Equation (19) takes the simpler view:

(20) $$\overset{↔}{\tilde{L}}\approx \left(1-\theta_{v}\right)\cdot \overset{↔}{\tilde{N}}$$

Therefore, the magnetic permeability tensor ${\overset{↔}{\mu }}^{m}$ of the composite medium with identically directed particles is determined by Expression (20), where the components of the tensor $\overset{↔}{\mu }$ are the same as in (19), but with replacement of the components of $\overset{↔}{\tilde{N}}$ by the components of $\overset{↔}{\tilde{L}}$. Finally, it can be written:

(21) $${\overset{↔}{\mu }}^{m}=\left(1-\theta_{v}\right)\cdot \overset{↔}{I}+\theta_{v}\cdot \overset{↔}{\mu }=\left(\begin{array}{ccc} \mu_{xx}^{m} & \mu_{xy}^{m} & 0\\ \mu_{yx}^{m} & \mu_{yy}^{m} & 0\\ 0 & 0 & 1 \end{array}\right)$$

$$\mu_{xx}^{m}=1-\theta_{v}+\theta_{v}\left[1+\frac{\omega_{M}{\left[\omega_{H}+i\omega \alpha -\left({\tilde{N}}_{zz}-{\tilde{N}}_{yy}\right)\;\left(1-\theta_{v}\right)\;\omega_{M}\right]}^{}}{\hat{D}}\right]$$

$$\mu_{xy}^{m}=\frac{\theta_{v}\omega_{M}{\left[i\omega -{\tilde{N}}_{xy}\left(1-\theta_{v}\right)\;\omega_{M}\right]}^{}}{\hat{D}}$$

$$\mu_{yx}^{m}=-\frac{\theta_{v}\omega_{M}{\left[i\omega +{\tilde{N}}_{xy}\left(1-\theta_{v}\right)\;\omega_{M}\right]}^{}}{\hat{D}}$$

$$\mu_{yy}^{m}=1-\theta_{v}+\theta_{v}\left[1+\frac{\omega_{M}{\left[\omega_{H}+i\omega \alpha -\left({\tilde{N}}_{zz}-{\tilde{N}}_{xx}\right)\;\left(1-\theta_{v}\right)\;\omega_{M}\right]}^{}}{\hat{D}}\right]$$

$$\hat{D}={\left[\omega_{H}+i\omega \alpha -\left({\tilde{N}}_{zz}-{\tilde{N}}_{xx}\right)\;\left(1-\theta_{v}\right)\;\omega_{M}\right]}^{}\left[\omega_{H}+i\omega \alpha -\left({\tilde{N}}_{zz}-{\tilde{N}}_{yy}\right)\;\left(1-\theta_{v}\right)\;\omega_{M}\right]-{\left({\tilde{N}}_{xy}\left(1-\theta_{v}\right)\;\omega_{M}\right)}^{2}-\omega^{2}$$

The foregoing formulas for the components of the magnetic permeability tensor are a generalization of formulas for a composite medium specified in [9]. They describe the resonance variations of permeability near FMR. Of course, these resonance features of frequency or magnetic field dependences of the component of the tensor (21) have to manifest themselves in the dependences of transmission and reflection coefficients and absorption of the wave. Besides, variations of non-resonant type are possible, which are caused by magnetization of the composite. These variations allow, for example, the so-called low field absorption [26]. In order to take into account non-resonant variations, the factor Χ is introduced, which corrects the values of the elements of the magnetic permeability tensor. One of the simplest ways to introduce Χ is the expression:

(22) $$Χ=1+\frac{\chi_{11}-1}{1+\kappa H_{z}^{2}}$$

where $\chi_{11}$ is the diagonal component of the susceptibility tensor at $H_{z}=0$. The contribution to dynamic magnetic permeability coming from the domain walls motion is calculated [27]. The magnetic field dependence of this contribution is very similar to our Equation (22). The components of the tensor ${\overset{↔}{\mu }}^{m}$ can be rewritten as:

(23) $$\begin{array}{l} \mu_{xx}^{m}=1+Χ\frac{\theta_{v}\omega_{M}{\left[\omega_{H}+i\omega \alpha -\left({\tilde{N}}_{zz}-{\tilde{N}}_{yy}\right)\;\left(1-\theta_{v}\right)\;\omega_{M}\right]}^{}}{\hat{D}},\\ \mu_{xy}^{m}=Χ\frac{\theta_{v}\omega_{M}{\left[i\omega -{\tilde{N}}_{xy}\left(1-\theta_{v}\right)\;\omega_{M}\right]}^{}}{\hat{D}},\\ \mu_{yx}^{m}=-Χ\frac{\theta_{v}\omega_{M}{\left[i\omega +{\tilde{N}}_{xy}\left(1-\theta_{v}\right)\;\omega_{M}\right]}^{}}{\hat{D}},\\ \mu_{yy}^{m}=1+Χ\frac{\theta_{v}\omega_{M}{\left[\omega_{H}+i\omega \alpha -\left({\tilde{N}}_{zz}-{\tilde{N}}_{xx}\right)\;\left(1-\theta_{v}\right)\;\omega_{M}\right]}^{}}{\hat{D}}. \end{array}$$

Notice that, according to (22), under condition $\chi_{11}$ =1, the equality Χ=1 is valid. The value of Χ is frequency dependent. Under magnetic saturation, at $1+\kappa H_{z}^{2}>>\chi_{11}-1$, Χ almost equals to 1.

#### 3.1.4. Magnetic Permeability of Ensemble of Arbitrarily Directed Particles

Let us set the vector of rotation angles of a ferromagnetic particle relative to the axes x, y, z as $\Theta =\left(\begin{array}{ccc} \alpha & \beta & \gamma \end{array}\right)$. It conditions, first, variation of the components of demagnetizing tensor $\overset{↔}{\tilde{N}}\left(\Theta \right)$ of the particle if its orientation varies and, second, variation of $M_{z}$, therefore, $\omega_{M}\left(\Theta \right)$. In order to get the effective magnetic permeability tensor $\langle {\overset{↔}{\mu }}^{m}\rangle$ of a composite medium in the case of arbitrarily oriented particles, it is necessary to perform the statistical averaging of its components:

(24) $$\langle {\overset{↔}{\mu }}^{m}\rangle =\left(1-\theta_{v}\right)\cdot \overset{↔}{I}+\theta_{v}\cdot \langle \overset{↔}{\mu }\rangle =\langle \left(\begin{array}{ccc} \mu_{xx}^{m}\left(\Theta \right) & \mu_{xy}^{m}\left(\Theta \right) & 0\\ \mu_{yx}^{m}\left(\Theta \right) & \mu_{yy}^{m}\left(\Theta \right) & 0\\ 0 & 0 & 1 \end{array}\right)\rangle$$

The effective magnetic permeability of the transversely magnetized medium $\mu_{eff}\left(\Theta \right)$, corresponding to a given value of the vector Θ, is expressed by a relation similar to (8), which includes the components of the tensor $\mu_{ij}^{m}\left(\Theta \right)$. The averaged value of the effective magnetic permeability for the transversely magnetized medium is determined by the formula:

(25) $$\langle \mu_{eff}\rangle =\langle \mu_{eff}\left(\Theta \right)\rangle =\langle \mu_{xx}^{m}\left(\Theta \right)-\frac{\mu_{xy}^{m}\left(\Theta \right)\cdot \mu_{yx}^{m}\left(\Theta \right)}{\mu_{yy}^{m}\left(\Theta \right)}\rangle$$

If particles have the form of flakes, their space orientation can be characterized by the direction of normal to its plane. If the normal is directed along to the *y* axis, then it can be defined by the vector $n=\left(\begin{array}{ccc} 0 & 1 & 0 \end{array}\right)$. Let us believe that this the initial or base orientation of a ferromagnetic particle, for which the vector of rotation angles can be defined as $\Theta_{0}=\left(\begin{array}{ccc} 0 & 0 & 0 \end{array}\right)$. All other possible space orientations of particles have to be determined by the vectors $\Theta =\left(\begin{array}{ccc} \alpha & \beta & \gamma \end{array}\right)$. In the case of arbitrary orientation of a great number of particles, every element from the multitude of vectors Θ belongs to the independent multitudes of uniformly distributed random numbers which get into the intervals: α∈[−π ; π], β∈[−π ; π] and γ∈[−π ; π]. The discrete number of the vector of rotation angles $\Theta_{p}$ corresponds to the discrete multitudes of numbers $\alpha_{p}$, $\beta_{p}$ and $\gamma_{p}$.

**Figure 4:**
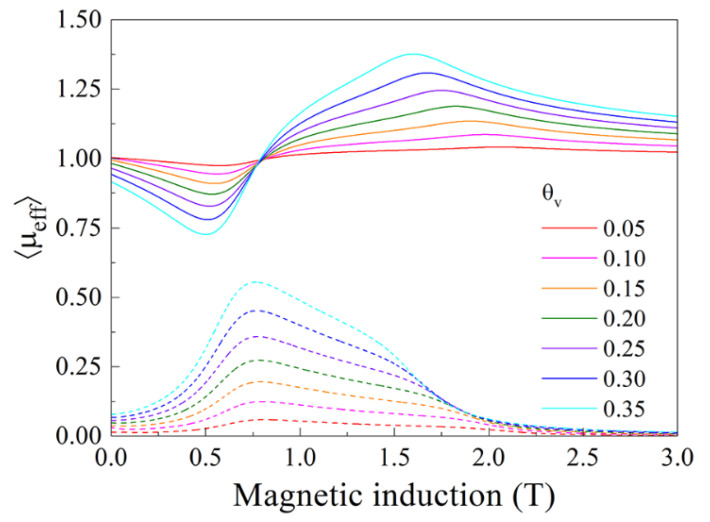
Calculated magnetic field dependence of the effective magnetic permeability for the composite containing 10,000 particles of material with the saturation magnetization *M_s_* = 900 kA/m and the magnetic damping constant *α* = 0.05. Every particle has the form of ellipsoid with the axes *a* = *b* = 25 µm and *c* = 1 µm. The calculation is carried out for frequency *f* = 32 GHz. The continuous lines correspond to the real part of permeability and the dashed lines correspond to the imaginary part of permeability.

The calculation of the effective magnetic permeability of a magnetized composite has been carried for 10,000 particles made of material with the saturation magnetization *M_s_* = 900 kA/m and magnetic damping constant *α* = 0.05. Every particle has the form of ellipsoid with the axes *a* = *b* = 25 µm and *c* = 1 µm. The calculation is carried out for frequency *f* = 32 GHz. The results for several values of $\theta_{v}$ are shown in Figure 4. The variations of the effective permeability caused by FMR are present in the graphs. It is evidently seen, from Figure 4, that the resonance occupied a wide region of magnetic fields despite the small value of *α*. Increase in the volume fraction of ferromagnetic particles leads to more pronounced resonance variations.

Let us consider the case when a group of particles with definite orientation is present in the composite besides the group of arbitrarily oriented particles. Denote the whole number of ferromagnetic particles as L; the number of arbitrarily oriented particles as $L_{1}$; the number of particles with the definite orientation (in the plane of a sample, for example) as $L_{2}$, $L=L_{1}+L_{2}$. In the limiting case when $L_{2}=0$, all particles are oriented arbitrarily, when $L_{2}=L$, all particles are oriented in the plane of a sample. The formula for calculation of the components of the tensor $\langle {\overset{↔}{\mu }}^{m}\rangle$ can be written for the presence of several groups of differently oriented particles. Their mean values are determined by the equation:

(26) $$\langle \mu_{eff}\rangle =\frac{L_{2}\left(\mu_{xx}^{m}\left(\Theta_{0}\right)-\frac{\mu_{xy}^{m}\left(\Theta_{0}\right)\cdot \mu_{yx}^{m}\left(\Theta_{0}\right)}{\mu_{yy}^{m}\left(\Theta_{0}\right)}\right)+\sum_{p=1}^{L_{1}}\left(\mu_{xx}^{m}\left(\Theta_{p}\right)-\frac{\mu_{xy}^{m}\left(\Theta_{p}\right)\cdot \mu_{yx}^{m}\left(\Theta_{p}\right)}{\mu_{yy}^{m}\left(\Theta_{p}\right)}\right)}{L_{1}+L_{2}}$$

**Figure 5:**
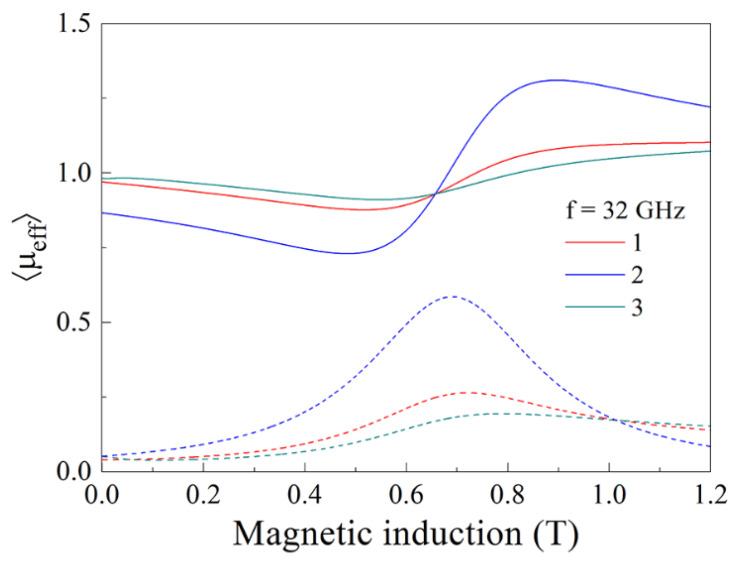
Magnetic field dependence of the effective magnetic permeability calculated for the composite with 5000 particles with the saturation magnetization of particles *M_s_* = 900 kA/m, the magnetic damping constant *α* = 0.19 and the volume fraction of ferromagnetic particles $\theta_{v}=0.15$. Curve 1 corresponds to the case where $\chi_{11}$ = 1, *L_1_* = 4000, *L_2_* = 1000. For curve 2, $\chi_{11}$ = 1 as well, but all particles are oriented in the plane of the sample and the DC magnetic field lies in the same plane. Curve 3 is calculated at $\chi_{11}$ = 1.4 and *κ* = 1.184 × 10^−9^ (m/A)^2^, that is, the non-resonant contribution in permeability is taken into account. The calculation is carried out for frequency *f* = 32 GHz. The continuous lines correspond to the real part of permeability and the dashed lines correspond to the imaginary part of permeability.

The results of the calculation of the effective magnetic permeability following to Equation (27) are shown in Figure 5 for the composite with 5000 particles of ellipsoidal form with the same sizes as in Figure 4. The saturation magnetization of particles is $M_{s}$ = 900 kA/m, the magnetic damping constant *α* = 0.19 and the volume fraction of ferromagnetic particles $\theta_{v}=0.15$. In Figure 5, curve 1 corresponds to the case $\chi_{11}$ = 1, that is, the non-resonant absorption is not taken into account. It is assumed, in this case, that *L_1_* = 4000, *L_2_* = 1000 and, namely, 20% particles are oriented by the manner when the DC magnetic field is in parallel to their plane. The remainder 80% particles are oriented arbitrarily. Curve 2 corresponds to the case when all particles are oriented to the plane of the sample and the DC magnetic field lies in this plane. The resonant-type variations are expressed much more strongly in this case. The values $\chi_{11}$ = 1.4 and *κ* = 1.184 × 10^−9^ (m/A)^2^ are chosen for curve 3, so the non-resonant contribution is taken into account. The calculation is carried out for frequency *f* = 32 GHz.

The results in Figure 4 and Figure 5 show that well-known procedures of homogenization based on the constitutive parameters and concentrations of its components are insufficient in order to describe the resonance phenomena in the heterogeneous magnetic medium; see [3] for example. The conception of the mean demagnetizing factor is sometimes used instead of the procedure of averaging of contributions in the components of the magnetic permeability tensor from particles with different demagnetizing factor [16]. This conception is capable, for instance, to describe the FMR spectrum of the composite material containing spherical particles [24]. For media in which the value of the demagnetizing factor of particles varies essentially, it is prescribed to apply the afore-mentioned calculation procedure. Notice that division of particles into separate groups with identical value of the demagnetizing factor used in our calculation in Figure 5 is similar to the method applied in [16].

### 3.2. Reflection, Transmission Coefficients and Dissipation of Microwaves

Let us discuss how the variations of magnetic permeability can disturb the quantities, which can be measured in an experiment. Let the electromagnetic wave of centimeter or millimeter wavebands fall from vacuum (that is medium 1) onto the plate of composite with thickness $d_{2}$ (that is medium 2). After the wave passes the plate, it enters the vacuum again. The complex transmission T and reflection R coefficients can be calculated via the equations [12,28]:

(27) $$T=\frac{2Z_{1}Z_{2}}{2Z_{1}Z_{2}\cos\;\left(k_{2}d_{2}\right)+i\;\left(Z_{1}^{2}+Z_{2}^{2}\right)\;\sin{\left(k_{2}d_{2}\right)}^{}{}^{}}$$

(28) $$R=\frac{i\;\left(Z_{2}^{2}-Z_{1}^{2}\right)\;\sin\left(k_{2}d_{2}\right)}{2Z_{1}Z_{2}\cos\;\left(k_{2}d_{2}\right)+i\;\left(Z_{1}^{2}+Z_{2}^{2}\right)\;\sin{\left(k_{2}d_{2}\right)}^{}{}^{}}$$

Here, $Z_{1}=\sqrt{\frac{\mu_{0}}{\varepsilon_{0}}}$ is the impedance of the medium 1, $Z_{2}=\sqrt{\frac{\mu_{0}\langle \mu_{eff}\rangle }{\varepsilon_{0}\varepsilon }}$ is the impedance of the composite with the dielectric permittivity $\varepsilon =\varepsilon^{\prime }-i\varepsilon^{\prime \prime }$ and the mean value of the effective magnetic permeability is $\langle \mu_{eff}\rangle ={\langle \mu_{eff}\rangle }^{\prime }-i{\langle \mu_{eff}\rangle }^{\prime \prime }$. Assume that the dielectric permittivity is a scalar value and it does not depend on the magnetic field. In Equations (27) and (28), $k_{2}={k^{\prime }}_{2}-i{k^{\prime \prime }}_{2}$ is the complex wavenumber which real and imaginary parts can be calculated, following to the formulas [9]:

$${k^{\prime }}_{2}=\frac{\omega }{c_{}}\sqrt{\frac{\left|\varepsilon \right|\;\left|\langle \mu_{eff}\rangle \right|+\varepsilon^{\prime }{\langle \mu_{eff}\rangle }^{\prime }-\varepsilon^{\prime \prime }{\langle \mu_{eff}\rangle }^{\prime \prime }}{2}}$$

$${k^{\prime \prime }}_{2}=\frac{\omega }{c_{}}\sqrt{\frac{\left|\varepsilon \right|\;\left|\langle \mu_{eff}\rangle \right|-\varepsilon^{\prime }{\langle \mu_{eff}\rangle }^{\prime }+\varepsilon^{\prime \prime }{\langle \mu_{eff}\rangle }^{\prime \prime }}{2}}$$

The power transmission $T_{P}$ and reflection $R_{P}$ coefficients can also be introduced. They show the portions of microwave power in the wave that passed the plate and was reflected from one:

(29) $$T_{P}=T\cdot T^{*},$$

(30) $$R_{P}=R\cdot R^{*}.$$

Dissipation of microwaves *D* can be calculated from these coefficients as follows:

(31) $$D=1-T_{P}-R_{P}.$$

Dissipation shows which fraction of the incident wave power is lost inside the plate. The losses come from absorption, scattering on the inner inhomogeneities and from transformation into the other waves besides TE_10_ at the sample boundaries. Several contributions, such as absorption, are magnetic field dependent but the others are not. It was seen, from Equations (27) and (28), that the peculiarities in the effective magnetic permeability coming from FMR, for example, also lead to variations of the transmission and reflection coefficients and wavenumber.

## 4. Microwave Results

**Figure 6:**
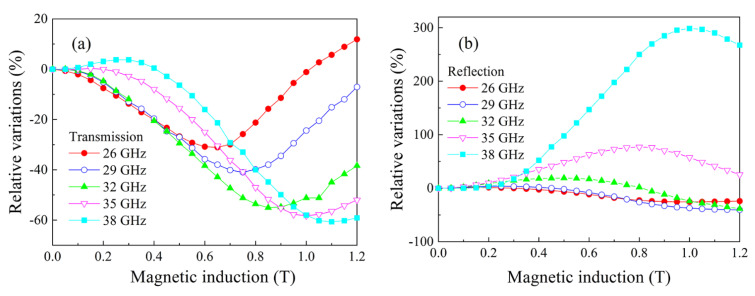
Magnetic field dependences of the transmission (**a**) and the reflection (**b**) coefficients for the composite with 30% flakes measured at several frequencies of millimeter waveband.

The results of the measurements show that the microwave transmission and reflection coefficients experience large variations under the action of a magnetic field. The results of measurements of the magnetic field dependences of the transmission and reflection coefficients for the composite with 30% flakes are shown in Figure 6. The measurements were performed at several frequencies of millimeter waveband. The characteristic feature of all the dependences in Figure 6a is the presence of a minimum. If frequency increases, then this minimum shifts to higher magnetic fields. The absolute value of the minimum increases if frequency increases. The minimum is caused by absorption of microwaves under FMR condition [13]. At the highest frequency of 38 GHz, a maximum also presents in the field of $\mu_{0}H$ = 0.35 T, i.e., less than the field of the minimum. As shown in [13], this maximum is caused by ferromagnetic antiresonance (FMAR). The results of the measurements of magnetic field dependence of the reflection coefficient in Figure 6b confirm the abovementioned statements. The minimum caused by FMR is presented in the dependences. The most distinctive feature of the reflection coefficient variations seems to be the maximum caused by FMAR. These variations reach 300% in magnitude; therefore, they could be regarded as giant ones.

**Figure 7:**
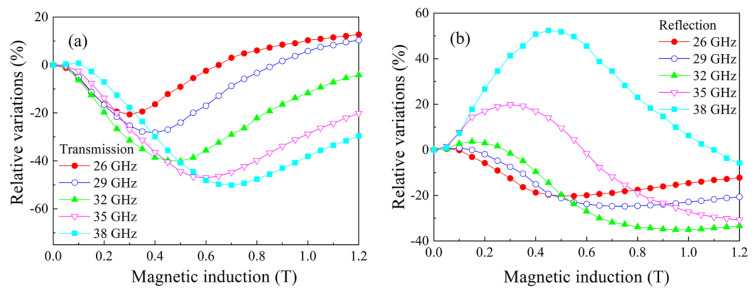
Magnetic field dependences of the transmission (**a**) and the reflection (**b**) coefficients for the composite with 15% flakes measured at several frequencies of millimeter waveband.

**Figure 8:**
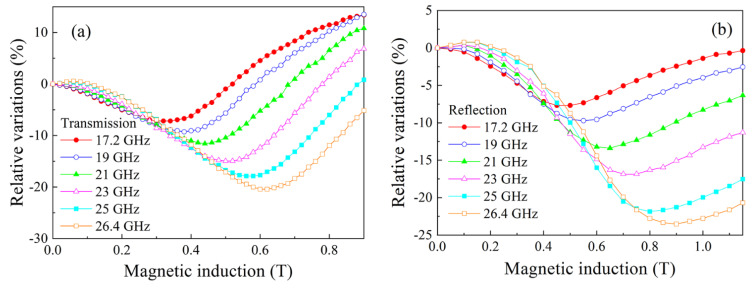
Magnetic field dependences of the transmission (**a**) and the reflection (**b**) coefficients for the composite with 15% flakes measured at several frequencies of centimeter waveband.

Analogous measurements were performed for the composite with 15% flakes and the results are shown in Figure 7. The minimum caused by FMR and the maximum caused by FMAR were also observed, but their magnitudes are less than in the previous case. The resonance variations were also observed at lower frequencies of centimeter waveband; see Figure 8. In this case, the fields of the minimum are less than in Figure 7, in accordance with the typical spectrum of the uniform FMR mode.

The weak maximum caused by FMAR, was observed only for frequency 26.4 GHz. Really, the FMAR phenomenon cannot present in frequencies less than some frequencies defined by the saturation magnetization [9]. Therefore, in the composites containing flakes of finemet-type alloy, the resonance variations are present, which were caused by both FMR and FMAR. For the composite with 30% flakes, the variations of the reflection coefficient under FMAR one can regard as giant ones.

## 5. Discussion

In this section, let us compare the experimental results with the theoretical calculations fulfilled before, in Section 3. We consider several versions of calculations, which differ from each other by the portion of flakes, the presence or, in contrast, absence, of the group flakes with identical space orientation and with taking into account, or lacking, the non-resonant variation of the dynamic magnetic permeability. In order to calculate the transmission and reflection coefficients, according to the Formulas (27) and (28), it is necessary to know the dielectric permittivity of the samples. The samples used in this paper are the same as in [29] and we take the values of permittivity from that work. The mean values of the dielectric permittivity for the three frequency ranges and the values of the microwave conductivity for the composite with 15% flakes are presented in Table 1. For the composite with 30% flakes in the frequency range from 26 to 38 GHz, the mean values are the following: *ε*′ = 38, *ε*″ = 13 and *σ* = 22 S/m.

**Figure 9:**
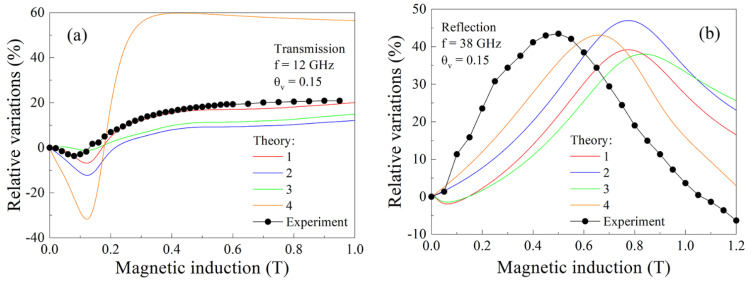
Relative variations of the transmission (**a**) and reflection (**b**) coefficients for the composite with 15% flakes. Designations of the lines correspond to the following parameters: 1—$\chi_{11}$ = 1.3, *L_1_* = 4000, *L_2_* = 1000; 2—$\chi_{11}$ = 1, *L_1_* = 4000, *L_2_* = 1000, 3—$\chi_{11}$ = 1.3, *L_1_* = 5000, *L_2_* = 0; 4—$\chi_{11}$ = 1, *L_1_* = 0, *L_2_* = 5000 and *κ* = 1.184·10^−9^ (m/A)^2^.

In Figure 9, the experimental dependences of the transmission and reflection coefficients for the composite with 15% flakes are shown by black line with symbols. The calculations are carried out using Formulas (27) and (28) and the magnetic permeability is calculated via Formula (26). The dependence, measured at frequency *f* = 12 GHz, corresponds fairly well to the dependence which calculated taking into account the non-resonant contribution in magnetic permeability ($\chi_{11}\neq 1$), the presence of the group of flakes oriented in parallel to the top surface ($L_{2}\neq 0$) and the group of flakes oriented arbitrarily. If frequency increases, the calculated dependences, however, differ from the experimental ones. For example, the results of the calculation of the reflection coefficient are shown in Figure 9b at the frequency of 38 GHz. The magnitude of variations in all variants of calculation satisfactorily corresponds to the experimental data, but the field of maximum differs substantially. The FMAR field is less than the FMR field, as expected, and it is located outside of the FMR line. Note that the magnetic field dependence of the coefficients at a fixed frequency is mainly defined by dependence of magnetic permeability. Comparing the foregoing results, it can be concluded that the method of calculation of magnetic permeability developed above describes fairly well the area of FMR resonance but gives worse fit for weaker fields. The probable reason for this discrepancy consists in the very rough approximation (22) of the non-resonant contribution in the magnetic field dependence of magnetic permeability.

**Figure 10:**
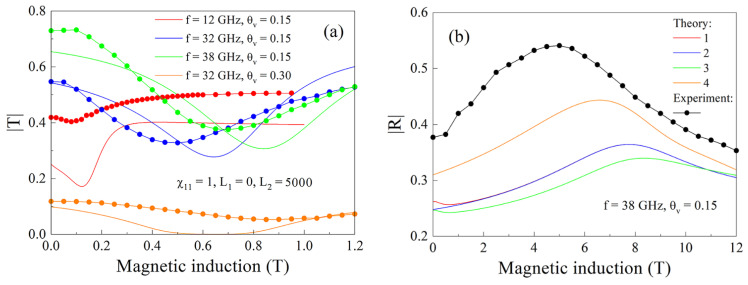
Magnetic field dependences of the transmission (**a**) and reflection (**b**) coefficients for the composites with 15% and 30% flakes. Designations of the lines: theory—continuous lines; experiment—lines with symbols. Designations of the lines in Figure (**b**) correspond to the following parameters: 1—$\chi_{11}$ = 1.3, *L_1_* = 4000, *L_2_* = 1000; 2—$\chi_{11}$ = 1, *L_1_* = 4000, *L_2_* = 1000, 3—$\chi_{11}$ = 1.3, *L_1_* = 5000, *L_2_* = 0; 4—$\chi_{11}$ = 1, *L_1_* = 0, *L_2_* = 5000 and *κ* = 1.184·10^−9^ (m/A)^2^.

The results presented in Figure 9 show the relative variations of the coefficients in the magnetic field. The absolute values of the coefficients are shown in Figure 10. The experimental data are compared to the calculations for the composite with $\theta_{v}=0.15$, for several frequencies, and for the composite with $\theta_{v}=0.3$, at the frequency of 32 GHz, in Figure 10a. In Figure 10b, the type of magnetic field dependence of the reflection coefficient and the presence of FMAR are reproduced in the calculation. From this fact, it can be concluded that qualitative correspondence is achieved between the experiment and calculation. The presence of the resonant variations is reproduced in calculation and the magnitudes of the resonant variations are reproduced for the composite with 15% flakes.

**Figure 11:**
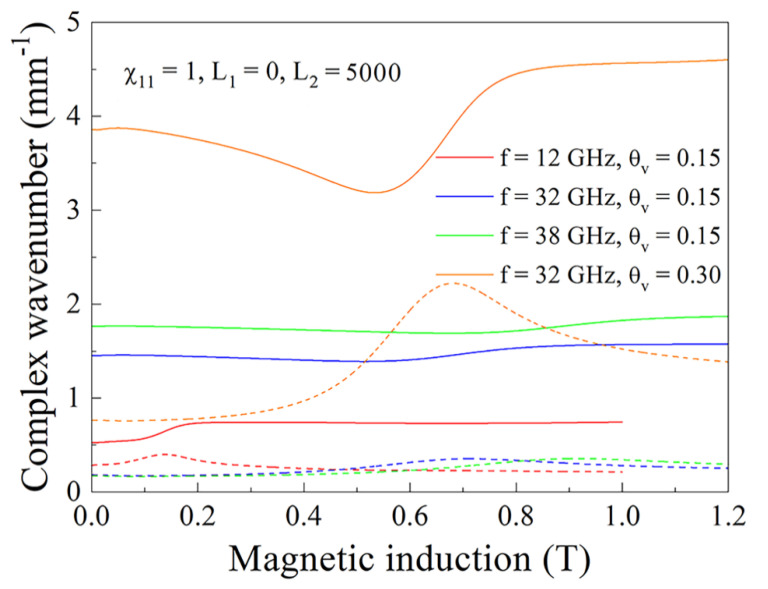
Real and imaginary parts of the wavenumber for the composites with 15% and 30% flakes at the waves of centimeter and millimeter wavebands. The continuous lines correspond to the real part of the wavenumber and the dashed lines correspond to the imaginary part of the wavenumber.

**Figure 12:**
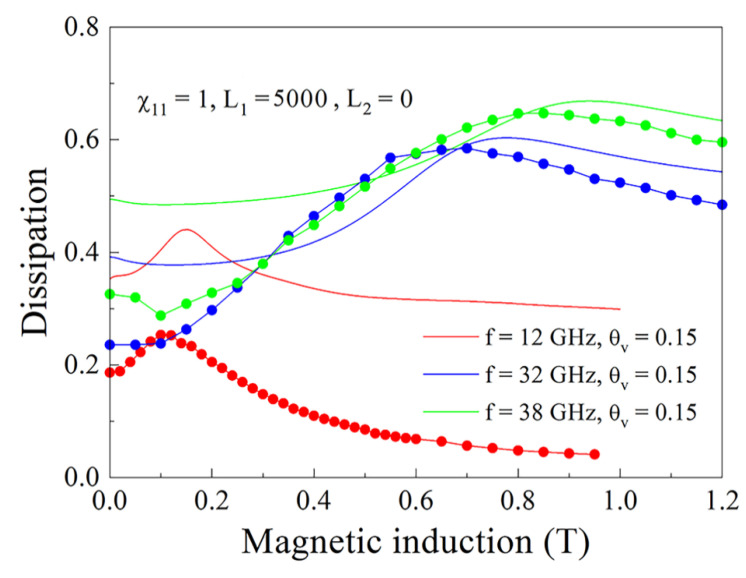
Magnetic field dependence of microwave dissipation for the composite with 15% flakes; calculation—the continuous lines; experiment—the lines with symbols.

In Figure 11 the magnetic field dependences of the real and imaginary parts of wavenumber for the same cases as presented in Figure 10a are shown. It is not surprising that the resonance variations are seen most brightly for the case $\theta_{v}=0.3$. In Figure 12, the magnetic field dependences of microwave dissipation determined via Formula (32) for the composite with $\theta_{v}=0.15$ are shown. Coincidence of the type of dependence and maximums caused by absorption under FMR condition occur both in calculation and in experiment. The absolute value of the dissipated power in calculation corresponds to experiment only in order of magnitude. Certainly, neither in experimental nor in calculated dependences, there are no peculiarities caused by FMAR.

**Figure 13:**
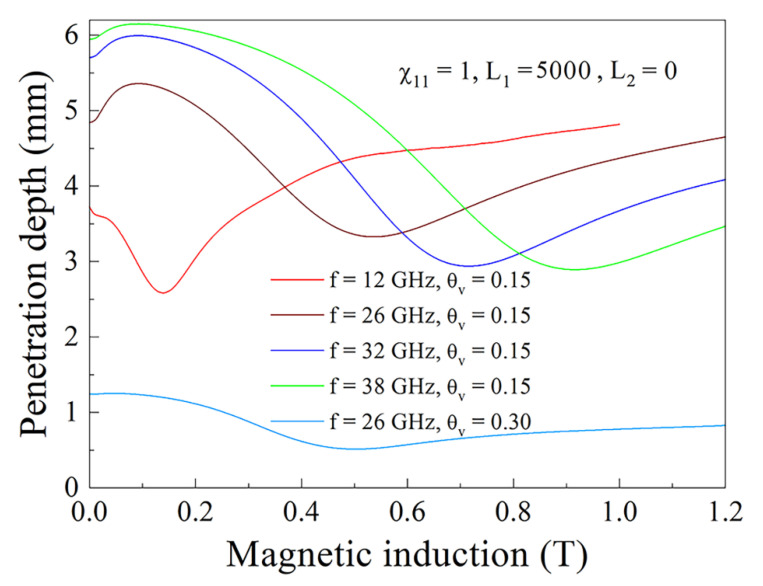
Magnetic field dependence of microwave penetration depth for the composites with 15% and 30% flakes.

The magnetic field dependence of the penetration depth of the electromagnetic field inside the composite sample is shown in Figure 13. The penetration depth, which is determined as $\delta =\frac{1}{{k^{\prime \prime }}_{2}}$, is introduced as the distance from the surface at which the electromagnetic field amplitude decreases by e times in comparison to that at the surface. In the case of metallic conductivity of a sample, *δ* is the skin depth. For the composite sample, which is a lossy dielectric, *δ* is the depth where electromagnetic field decreases because of absorption and scattering. This clarification is essential, because the composites with ferromagnetic flakes demonstrate properties of lossy dielectric in zero magnetic field as well as in fields that are significantly less than the field of FMR. Near the area of FMR, the real and imaginary parts of the wavenumber become the same order of magnitude that is characteristic for metals [13]. The dependences shown in Figure 13 once more confirm the fact that the resonance variations are caused by FMAR and FMR. Actually, at frequencies of 26 GHz and higher, if the magnetic field increases, a maximum of the penetration depth occurs that is typical for FMAR. A further increase of the magnetic field leads to a decrease and minimum of the penetration depth due to absorption of the waves under FMR. At frequencies less than 26 GHz, there is no maximum of *δ*, as expected for magnetic antiresonance. As expected, the penetration depth for the composite with 30% flakes is less than the same for the composite with 15% flakes.

## 6. Conclusions

The transmission of microwaves through the composite medium containing metallic ferromagnetic particles is discussed and so is the reflection from this medium. The theory is worked out which permits to calculate the components of the tensor of magnetic permeability for the composite containing the aggregate of particles in the form of ellipsoid the axes of which are directed in an arbitrary manner in relation to the coordinate axes. The expression is obtained for the calculation of the effective magnetic permeability of transversely magnetized composite medium, which contains both randomly oriented particles and a group of particles with a preferential direction. The calculation is carried out for magnetic field dependency of the effective permeability, which shows that, in the case of random orientation, a range of magnetic fields occurs, where resonant-type variations are realized, instead of a single FMR line.

The experiments are carried out with the composites containing 15% and 30% flakes of Fe-Si-Nb-Cu-B alloy. The microwave measurements are performed at frequencies from 12 to 38 GHz. The magnetic field dependences are measured for the transmission and reflection coefficients moduli. The peculiarities of resonance type are observed connected with absorption of microwaves under FMR condition. For frequencies above 26 GHz, the peculiarities caused by FMAR are also observed. For the composite with 30% flakes at the frequency of 38 GHz, in the region of FMAR, the reflection coefficient maximum is observed reaching 300%. Note that ferromagnetic antiresonance is observed in the composite medium which has no DC conductivity.

The calculations have been performed to allow a comparison between the calculated magnetic field dependences of the transmission and reflection coefficients and the measured ones. The calculations are carried out for several frequencies of microwaves and for several cases of orientation of flakes—for a group of particles with a preferential direction and for random orientation of particles. The non-resonant contribution into the magnetic permeability is taken into consideration. It has been shown that the calculation reproduces the presence of FMR and FMAR resonances and the type of magnetic field dependence of coefficients is presented correctly. An approximate quantitative correspondence is obtained for the magnitude of the resonance variations of the transmission coefficient under FMR condition. For magnetic fields that are less than the field of FMR, where FMAR is realized, qualitative correspondence is reached between experiment and theory. The microwave power is calculated and it is shown that a maximum of dissipation occurs near FMR. In the region of FMAR, there is no strong variation of the dissipation, neither in calculation, nor in experiment. The magnetic field dependence of the penetration depth of electromagnetic waves is calculated and it is shown that an increase in depth is realized near FMAR and a decrease near FMR.

## Figures and Tables

**Table 1:** Dielectric permittivity and microwave conductivity of samples.

Frequency Range (GHz)	Sample	*ε*′	*ε*″	*σ* (S/m)
12–18	Composite 15%	7.5	3.13	2.45
18–26	Composite 15%	8.2	1.5	1.9
26–38	Composite 15%	5.4	1.1	2.0

## Data Availability

Not applicable.
